# Low Prevalence of Ocular *Chlamydia trachomatis* Infection and Active Trachoma in the Western Division of Fiji

**DOI:** 10.1371/journal.pntd.0004798

**Published:** 2016-07-12

**Authors:** Colin K. Macleod, Robert Butcher, Umesh Mudaliar, Kinisimere Natutusau, Alexandre L. Pavluck, Rebecca Willis, Neal Alexander, David C. W. Mabey, Luisa Cikamatana, Mike Kama, Eric Rafai, Chrissy H. Roberts, Anthony W. Solomon

**Affiliations:** 1 Clinical Research Department, London School of Hygiene & Tropical Medicine, London, United Kingdom; 2 Sightsavers, Haywards Heath, United Kingdom; 3 Ophthalmology Department, Lautoka Hospital, Lautoka, Fiji; 4 Taskforce for Global Health, Atlanta, Georgia, United States of America; 5 MRC Tropical Epidemiology Group, Department of Infectious Disease Epidemiology, Faculty of Epidemiology and Population Health, London School of Hygiene & Tropical Medicine, London, United Kingdom; 6 Fiji Centre for Communicable Disease, Ministry of Health, Suva, Fiji; Alfaisal University, SAUDI ARABIA

## Abstract

**Background:**

Trachoma is the leading infectious cause of blindness and is caused by ocular infection with the bacterium *Chlamydia trachomatis* (Ct). While the majority of the global disease burden is found in sub-Saharan Africa, the Western Pacific Region has been identified as trachoma endemic. Population surveys carried out throughout Fiji have shown an abundance of both clinically active trachoma and trachomatous trichiasis in all divisions. This finding is at odds with the clinical experience of local healthcare workers who do not consider trachoma to be highly prevalent. We aimed to determine whether conjunctival infection with Ct could be detected in one administrative division of Fiji.

**Methods:**

A population-based survey of 2306 individuals was conducted using the Global Trachoma Mapping Project methodology. Population prevalence of active trachoma in children and trichiasis in adults was estimated using the World Health Organization simplified grading system. Conjunctival swabs were collected from 1009 children aged 1–9 years. DNA from swabs was tested for the presence of the Ct plasmid and human endogenous control.

**Results:**

The prevalence of active trachoma in 1–9 year olds was 3.4%. The age-adjusted prevalence was 2.8% (95% CI: 1.4–4.3%). The unadjusted prevalence of ocular Ct infection in 1–9 year-olds was 1.9% (19/1009), and the age-adjusted infection prevalence was 2.3% (95% CI: 0.4–2.5%). The median DNA load was 41 Ct plasmid copies per swab (min 20, first quartile 32, mean 6665, third quartile 161, max 86354). There was no association between current infection and follicular trachoma. No cases of trachomatous trichiasis were identified.

**Discussion:**

The Western Division of Fiji has a low prevalence of clinical trachoma. Ocular Ct infections were observed, but they were predominantly low load infections and were not correlated with clinical signs. Our study data suggest that trachoma does not meet the WHO definition of a public health problem in this Division of Fiji, but the inconsistency with previous studies warrants further investigation.

## Author Summary

Trachoma, caused by ocular strains of *Chlamydia trachomatis*, represents a major global public health issue, and is the subject of an international elimination campaign. Until recently, data on trachoma in the Pacific Island states have been sparse. The most recent studies have conflicted in their estimates of trachomatous disease burden in Fiji, therefore, surveys using alternative markers (infection testing plus grading) to those already used (grading alone) are warranted to try to shed further light on trachoma epidemiology in this setting. We used an externally validated clinical assessment protocol to show that evidence of active trachoma is present at a low prevalence, and we did not find any cases of trichiasis, the sight-threatening stage of trachoma. From testing of conjunctival swabs with a validated, next-generation PCR, we also found that *C*. *trachomatis* was present at a low prevalence. Our clinical data suggest that trachoma does not meet the WHO definition of a public health problem in this Division of Fiji, but the inconsistency with previous studies warrants further investigation.

## Introduction

Trachoma is the leading infectious cause of blindness, and is caused by ocular infection with the bacterium *Chlamydia trachomatis* (Ct). Trachoma is thought to be a public health problem in 51 countries, with 232 million people at risk of blinding disease [1]. Infection may present as an acute and self-limiting keratoconjunctivitis, but numerous re-infections can lead to potentially blinding sequelae.

Globally, the highest prevalence of active trachoma is found in sub-Saharan Africa [1]. Transmission of infection is thought to be through direct contact with hands or cloths which transfer ocular or nasal discharge between individuals [2], although the bacteria can also be spread by passive contact with eye-seeking *Musca sorbens* flies [3].

Trachoma is diagnosed by clinical examination of the eye. Active trachoma is characterised by the presence of 5 or more >0.5mm lymphoid follicles in the central part of the upper tarsal conjunctiva (trachomatous inflammation–follicular, TF) and/or pronounced inflammatory thickening of the upper tarsal conjunctiva obscuring more than half the normal deep tarsal vessels (trachomatous inflammation-intense, TI) [4]. Scar tissue deposited during resolution of inflammatory disease episodes leads, in some individuals to the misdirection of eyelashes so that they touch the eyeball; this state is known as trachomatous trichiasis (TT) [4,5].

The World Health Organization (WHO) advocates the use of the SAFE strategy–Surgery for trichiasis, Antibiotics, Facial cleanliness, and Environmental improvement, for elimination. Annual mass drug administration (MDA) of the antibiotic azithromycin is recommended for at least 3 years in any district where the prevalence of TF in 1–9 year olds is estimated to be 10% or greater. The decision to undertake MDA is informed by data from a population-based prevalence survey (PBPS) [6] in any district that has been identified as being of concern. The Global Trachoma Mapping Project (GTMP) [7] is currently undertaking PBPSs in all secure probably-endemic districts worldwide, in an effort to complete the baseline trachoma map by the end of 2015.

Cases of trachoma have historically been reported in Fiji [8–11]. A 2007 rapid assessment found a high prevalence of active trachoma in targeted Fijian villages, but no cases of TT [12]. In 2012, a PBPS was undertaken in each of Fiji’s four divisions, which estimated division-level prevalences of TF in 1–9 year olds ranging from 10.4–20.9% (19.6% in Western Division). Individuals aged over 15 years were examined only in Western and Northern Divisions, with prevalences of TT in that age group estimated at 8.7% and 6.2%, respectively [13]. The 2012 PBPS results suggested that trachoma was highly endemic in Fiji, and that the prevalence of TT was among the highest in the world. This was in stark contrast to the experience of Fijian ophthalmologists who see cases of TT quite infrequently [12–14].

**Fig 1:**
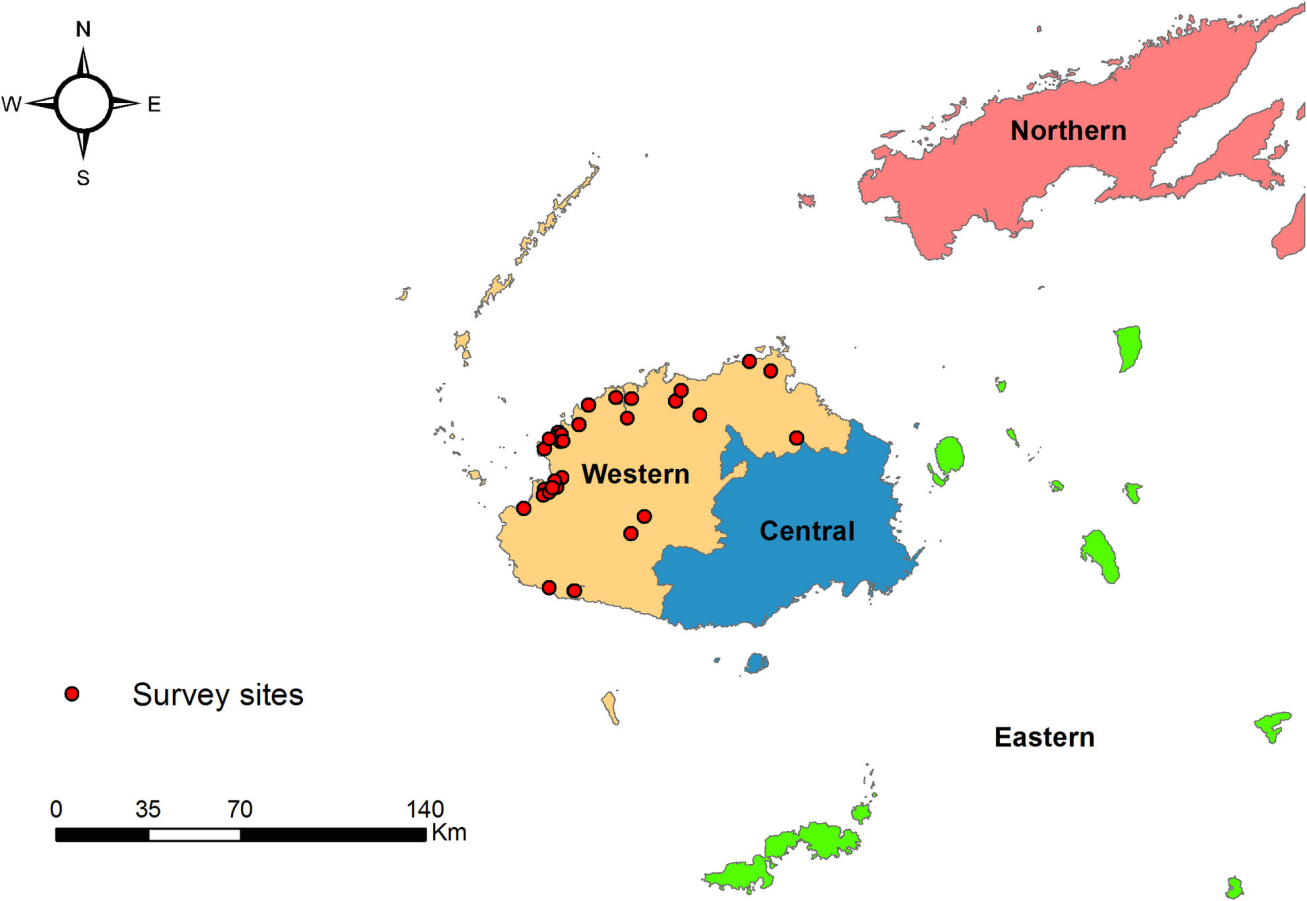
The four administrative divisions of Fiji, with the selected clusters in the Western division marked as points. Prepared using ArcGIS 10.2 (ESRI).

We conducted a PBPS for TF and TT in the Western Division of Fiji (total rural population 184,039; Fig 1) [15]. In addition we collected conjunctival swabs from children, which were then processed and subjected to PCR with the aim of estimating the prevalence of ocular Ct infection.

## Methods

### Ethics statement

The study was conducted in accordance with the Declaration of Helsinki. Consent was obtained from the leader of each community prior to entry into the community. Where possible, village chiefs, local headmen or local leaders were contacted in advance of the survey to gain consent to enter the respective villages. In indigenous Fijian villages, sevu-sevu, a traditional welcome ceremony involving sharing a Kava root-infused water with village leaders, was performed in accordance with the local custom. The study was designed to be paper-free which enabled real-time data upload and review, and streamlined field logistics. In this rural Fijian context, it was considered culturally appropriate for those over the age of 15 to consent for themselves. Verbal informed consent to be examined was obtained from each participant over the age of 15 years. For participants under the age of 15 years, consent for examination and to have specimens collected was given on their behalf by a legally responsible parent or guardian. The Fiji National Research Ethics Review Committee and the London School of Hygiene & Tropical Medicine ethics committee approved this consent protocol. All consent was recorded electronically via the Android phone-based data-capture system [7].

### Study design

A cross-sectional, cluster random sample survey methodology was conducted in November and December 2013. Villages identified from local census lists were considered as potential clusters in the sampling. A total 31 villages were selected for inclusion, with 30 households sampled per cluster. The total number of villages was calculated based on the anticipated number of children per household from the latest available census data [15]. In the first stage, after the exclusion of urban centres, villages were sampled with probability proportional to size. At the second stage of sampling, 30 households within a village were selected. Households were selected at random on the day of survey from a list of village households compiled by the village health worker or the village leader. Any person aged one year or more living in a sampled households was invited to participate. Effort was made to ensure participation by absent household members by returning later in the day where possible.

### Sample size

The study was powered to estimate a 10% prevalence of ocular Ct infection in 1–9 year olds with absolute precision of ±3% and 95% confidence. A design effect correction of 2.65 was used, based on previous trachoma surveys [7]. We included 10% oversampling in order to account for non-response, the required sample size was 1120 children in this age group. Based on 2007 census data, we expected to find 1.2 children aged 1–9 years per household, therefore we estimated 30 households from each of 31 clusters would be sufficient to recruit 1120 children. The overall sampling methodology was in accordance with that used in the GTMP [7].

### Data collection

Data were collected on an Android smartphone using a slightly modified version of the GTMP LINKS app, which is an implementation of the Open Data Kit toolbox for mobile data collection efforts (https://opendatakit.org/) and has been described elsewhere [7]. GPS coordinates were recorded for each participating household. The age and sex of each household member was then recorded, along with a record of consent to examination, refusal or absence at the time of the survey.

### Clinical assessment

A single GTMP-certified [7] trachoma grader examined both eyes of each participant using a 2.5× binocular loupe and sunlight. Each eye was assessed for the presence or absence of TT, TF and TI, according to the WHO simplified grading system [4,7]. An individual trained in the use of the Android phone application recorded results. Disposable gloves were used during swab collection, and alcohol hand gel was used between individuals to prevent carry-over contamination from one subject to the next. Participants found to have active trachoma were provided with a course of 1% tetracycline ointment and directions in its method of application. Participants found to have any significant ocular pathology were referred to the nearest eye care centre for management.

### Conjunctival sampling

For each participating child aged 1–9 years, a specimen was taken from the right upper tarsal conjunctiva with a polyester swab (Puritan Medical Products, ME, USA) and using a standardised collection procedure [16]. The specimen was taken immediately after clinical grading and the swab was immediately returned to its packet, secured and labelled with an anonymised five-digit numeric code. Swabs were kept in the field in a cool, dry container and were then air-dried overnight, before being transferred to 5°C storage the following morning; they were then maintained at this temperature until processing, between 1 and 5 months later.

### Control swabs

Fifteen negative field control swabs were collected by passing a swab within 15 cm of the eyes of a seated subject, chosen by random selection from the list of all specimen labels prior to commencement of the survey; specimen labels were used sequentially. Positive control swabs were prepared by briefly submerging swab heads in an homogenized solution of Ct strain A2497[17] elementary bodies and cultured hep2C cells at a dilution factor of 1 in 500, suspended in a phosphate-buffered saline. 15 such positive control swabs were prepared in London and stored in 2 mL Eppendorf tubes, then frozen and retained at LSHTM, UK. 15 further positive controls were prepared in the field, and were stored at 5°C until analysis. Field control swabs and swabs from study subjects were indistinguishable, and laboratory staff were masked to swab status.

### DNA extraction

Genomic DNA was extracted in to 50 μL nuclease free water using the Norgen Genomic DNA Purification kit (Norgen Biotek, Canada) according to manufacturer's protocol. For quality control, a sample with DNA extracted from a clean swab was included in each extraction batch.

### *C*. *trachomatis* infection testing

A Ct-specific droplet digital PCR (ddPCR) assay was used according to a published protocol[18], and with the minor modification that an 8 μL aliquot of DNA was used in each reaction. Briefly, each well contained 1X ddPCR supermix, 0.2 μM fluorescent probes and 0.9 μM forward and reverse primers for *Homo sapiens RPP30* and Ct plasmid ORF 2. Thermal cycling conditions were 95°C for 10 minutes, followed by 40 cycles of 95°C for 15 seconds and 60°C for 1 minute; then a final hold for 10 minutes at 98°C. A modified *omcB* probe was used to improve quenching efficiency and limit background fluorescence (Table 1).

**Table 1:** Primer and probe sequences for *C*.*trachomatis* targets and control using ddPCR. [19]

Molecular target and primer or probe	Nucleotide sequence and modifications
*Homo sapiens* RNase P/MRP 30-kDa	
subunit (RPP30) (endogenous control)	
RPP30-F	5ʹ AGA TTT GGA CCT GCG AGC G 3ʹ
RPP30-R	5ʹ GAG CGG CTG TCT CCA CAA GT 3ʹ
RPP30_HEX_BHQ1	5ʹ HEX-TTC TGA CCT GAA GGC TCT GCG CG-BHQ1 3ʹ
*C*. *trachomatis* cryptic plasmid	
pLGV440 (circular; genomic DNA; 7,500 bp)	
Ct-plasmid-F	5ʹ CAG CTT GTA GTC CTG CTT GAG AGA 3’
Ct-plasmid-R	5ʹ CAA GAG TAC ATC GGT CAA CGA AGA 3’
Ct-plasmid_FAM_BHQ1^b^	5ʹ 6FAM-CCC CAC CAT TTT TCC GGA GCG A-BHQ1 3ʹ
Ct-plasmid_HEX_BHQ1^c^	5ʹ HEX-CCC CAC CAT TTT TCC GGA GCG A-BHQ1 3ʹ
*C*. *trachomatis* (serovar A) *omcB* gene	
Ct-*omcB*-F	5ʹ GAC ACC AAA GCG AAA GAC AAC AC 3ʹ
Ct-*omcB*-R	5ʹ ACT CAT GAA CCG GAG CAA CCT 3ʹ
Ct-*omcB*-FAM-BHQ1	5ʹ 6FAM-CCA CAG CAA AGA GAC TCC CGT AGA CCG-BHQ1 3ʹ

*a* MRP, mitochondrial RNA processing endoribonuclease; 6FAM, 6-carboxyfluorescein reporter; BHQ1, black hole quencher 1; HEX, hexachlorofluorescein reporter.

*b C*. *trachomatis* plasmid probe used in screening (first) assay.

*c C*. *trachomatis* probe used in quantitative (second) assay.

Specimens from persons with TF and/or TI were tested for the presence of Ct *omcB*, a well-conserved genomic target, to ensure that cases of infection were not missed due to insertion/deletion or recombination events disrupting the site of the diagnostic primers for plasmid DNA [20].The number of plasmids per chromosome was also assessed using the method described by Last et al [19]. A single well was run for each sample.

### Statistical analysis

Data analysis was carried out using R [21]. Observed cluster-level frequencies of TF were adjusted for age in one-year age-bands using data from the 2007 Fiji census [7,15]. Confidence intervals were calculated by bootstrapping adjusted cluster-level proportions [22]. A binomial confidence interval was used for the upper bound of the TT prevalence estimate [23]. ddPCR data were analysed using QuantaSoft software (BioRad, Hemel Hempstead, UK). A positive ddPCR result was defined as one having a greater than 95% confidence in a non-zero load under a Poisson approximation, as described elsewhere [18].

## Results

### Descriptive epidemiology

A total of 413 households were visited over 31 clusters. No data were collected on non-participation of households. We enumerated 2306 individuals for inclusion in the study, of whom 1038 were aged 1–9 years, 335 were aged 10–14 years and 933 were aged 15 years and over. Ten (0.4%) individuals declined consent to participate; 2296 were examined. The median age of those examined was 11 years (mean: 20; min: 1; 1st quartile: 5; 3rd quartile: 32; max: 91), and 1289 (56.1%) were female. Following data cleaning, 1009 children were included in the study, which is very close to the targeted sample size of 1018.

### Clinical assessment

Data records without paired clinical and swab data (n = 29/1038) were discarded from the analysis. TF was observed in 34/1009 (3.4%) 1–9 year-olds. The age-adjusted prevalence of TF was 2.8% (95% CI 1.4–4.3%;). TI was observed in 2/1009 (0.2%) 1–9 year-olds. The age-adjusted prevalence of TI was 0.1% (95% CI: 0.0–0.3). No cases of TT were observed in 928 examined participants aged 15 and over. The age-adjusted prevalence of TT in those aged 15 years and above was 0% (95% CI 0–0.2%).

In addition to the 34 cases of TF found in those aged 1–9 years, 6.4% (21/330) of those aged 10–14 years and 1.1% (10/928) of those aged 15 years and over were found to have clinical signs of TF and/or TI. The median age of those with TF was 8 years (mean: 11; min: 1; first quartile: 5; third quartile: 11; max: 83).

### Ocular *C*. *trachomatis* infection

A total of 1038 children aged 1–9 years had ocular swabs taken and analysed. 16 (1.5%) swabs were unusable due to labelling errors. Of the 1022 remaining, 13 (1.3%) failed quality control because there was no detectable endogenous human target. 1009 (97.1%) specimens passed quality control (>95% confidence in non-zero human *RPP30* load). The mean droplet number per well was 14062 (first quartile 12634, median 13837, third quartile: 15247). The median endogenous control load was 9576 *RPP30* copies/swab. 19/1009 (1.9%) tested positive for the presence of Ct plasmid DNA. 1/34 (3.1%) children with active disease tested positive for Ct DNA and 18/977 (1.8%) children without active disease tested positive (Table 2). There was no association between cases of TF and cases of infection (p = 0.644 Mantel-Haenszel Chi-square). No new cases of infection were detected when active disease cases were retested with a multiplex plasmid/*omcB* ddPCR assay.

**Table 2:** Qualitative test for infection in persons with and without clinically active trachoma.

	TF/TI absent	TF/TI present	Total
	(n, %)	(n, %)	
**ddPCR–ve**	957 (98.2)	33 (97.1)	989
**ddPCR +ve**	18 (1.8)	1 (2.9)	19
**Total**	975 (100)	34(100)	1009

The median load of infection in positive specimens was 41 Ct plasmid copies/swab (min 20, first quartile 32, mean 6665, third quartile 161, max 86354). Among the 19 swabs that tested positive for the plasmid, seven additionally tested positive for presence of *omcB* in the multiplexed plasmid/*omcB* test. The mean plasmid:chromosome ratio was 4.4 plasmids per chromosome (range 1–12), consistent with the findings of Last et al [19]. The age-adjusted prevalence of ocular Ct infection in 1–9 year-olds was 2.3% (95% CI: 0.4–2.5%).

### Control swabs

**Fig 2:**
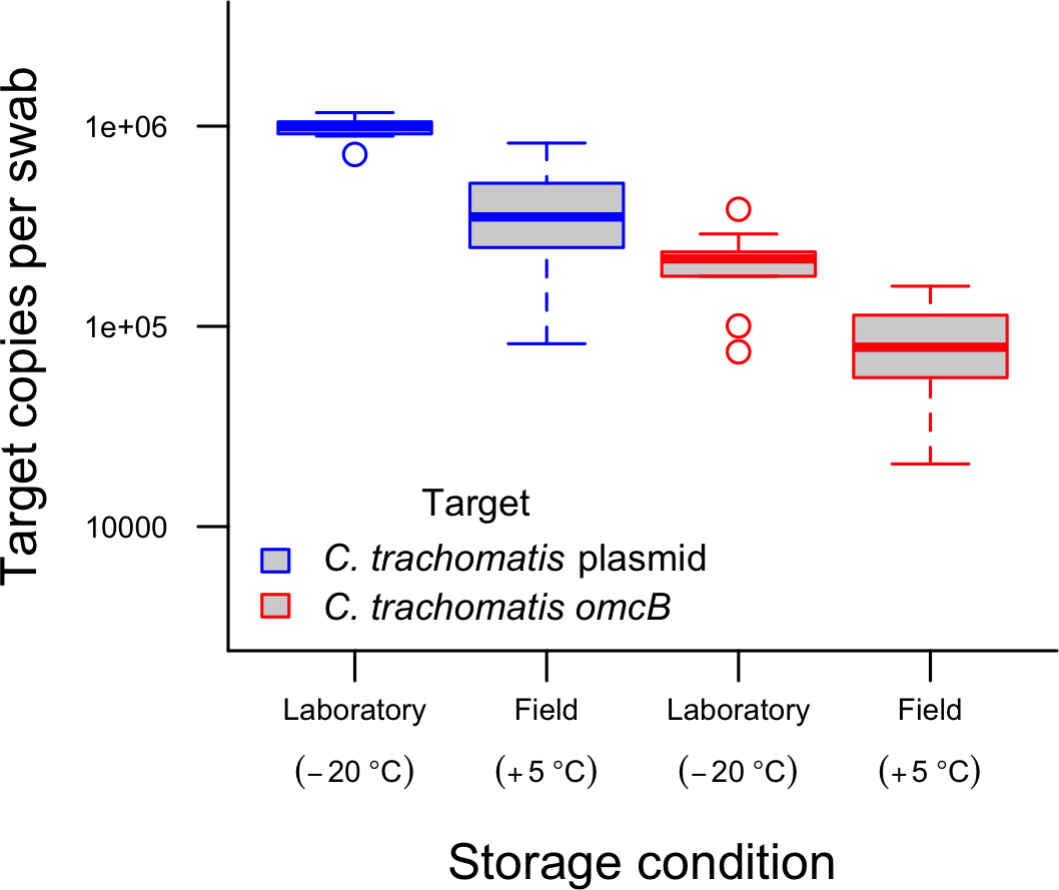
Comparative Ct *omcB* and plasmid load recovered from swabs stored at -20°C (n = 15) and +5°C (n = 15) for the duration of the study (November 2013 –July 2014).

All 30 positive control swabs (15 field and 15 lab) tested positive for Ct. The field control swabs had a 58.7% reduction in mean Ct plasmid load as compared to those stored frozen. Mean Ct *omcB* load for the field storage group was also reduced by 58.7%. The reductions in mean load of both plasmid and *omc*B were statistically significant (Pearson’s Chi-squared test p = 0.0002 and 0.000016 for difference in plasmid and *omcB*, respectively)). The mean load of human DNA from hep2C cells was 45% lower in the frozen swabs than in the swabs that were stored in the field, but this difference was not statistically significant (80 vs 148 copies/swab; p = 0.18) (Fig 2). Of the 15 negative control swabs collected in the field, 1 swab was lost in transit. The other 14 negative control swabs tested negative for both human and Ct DNA.

## Discussion

We estimate there to be a low prevalence of active trachoma and a correspondingly low prevalence of ocular Ct infection in the Western Division of Fiji. These data represent the first data on ocular Ct infection in the Pacific Island small states and represent a significant step towards improving knowledge of trachoma in the region.

We found no association between infection and active trachoma. This result is unsurprising, given the generally low correlation between signs of disease and ocular Ct infection in low prevalence settings [24–27]. It has been suggested that this may be because where Ct prevalence is low, other pathogens may be associated with the active trachoma phenotype [27,28]. It is possible that nucleic acid amplification testing may miss some low-load Ct positive samples due to the relatively high sampling variation when an analyte is at very low concentrations. Some commentators have suggested the sensitivity of this ddPCR assay may be too low for trachoma programs [29] due to the sensitivity observed in a ‘face value’ diagnostic evaluation by Roberts and colleagues. However, it was demonstrated that the discrepant results occurred in a mathematically predictable manner related to the analyte concentration and that most PCR-based technology will share an absolute limit to the number of analyte copies per test that will be reproducibly detected. It was highlighted that in a traditional discrepant analysis the sensitivity of this ddPCR assay could have been as high as 98% [30] and we therefore believe the test was appropriate in this setting.

For logistical reasons, swabs that were collected during this survey were not frozen during storage. A number of studies have illustrated that host [31] and chlamydial DNA [32,33] are stable in the short term when stored dry. Evidence from our positive control swabs indicates that a substantial proportion of chlamydial and human DNA is lost during storage over a few months at 5°C. However, this will only result in loss of qualitative diagnostic accuracy at very low loads of infection, consistent with the findings of Dize and colleagues [34]. In our positive control swabs, the ratio of plasmid to chromosome targets was the same regardless of storage conditions, indicating a similar rate of degradation between both genome components. We described above the difficulties caused by sampling error when diagnosing very low load infections, and degradation during storage may have caused previously detectable samples to become not reproducibly detectable. Improving specimen transport and storage conditions may have resulted in a closer association between clinical signs of disease and Ct infection. However, as low-load infections may be poorly associated with TF [16] and of limited importance in driving transmission at community level [35], we consider their detection not to be critical. The loss of sensitivity is however a limitation when considering the findings of this cross-sectional prevalence study.

We did not find any cases of TT in this population. This is in contrast to the previous (2012) PBPS, which found a high prevalence of TF (19.6% in 1–9 year-olds) and TT (8.7% in ≥15 year-olds) in this Division [13,36]. A 2009 rapid assessment found communities in which a high proportion of examined children had TF, but–like the present work—no cases of TT [12]. The source of these discrepancies is the subject of on-going research but we have observed social practices of eyelash epilation in Fiji that may have been misdiagnosed as trachomatous trichiasis.

The number of children per household was higher in this survey than in the 2007 national census, and we therefore reached our sample size in a lower number of households than expected. Data were not collected on households not enrolled in the study, nor on household demographics that may have explained why our sample size was reached with fewer houses than originally thought. However, the target sample size was very nearly achieved, and our individual participation rate was over 99%, therefore the risk of attrition bias is considered to be low. The difference in TF prevalence between the present data and the 2012 PBPS could be due to poor consensus between graders, seasonal variation in trachoma prevalence or an artefact of the cross-sectional study designs. The 2012 survey followed a PBPS protocol with random selection at village and household level with a comparable number of children sampled overall. The survey presented here sampled lower numbers of villages and households, but this is unlikely to sufficiently explain the large difference between the resultant TF prevalences found. Specific information on which villages were surveyed in the earlier study was not available, therefore it is not clear whether there was overlap between clusters visited; our randomisation process may have missed trachoma hotspots in Western Division by chance. The low estimate of Ct infection prevalence in this study does not support the estimated prevalence of TF observed in the previous survey, and could be a result of the relative non-specificity of phenotypic markers in trachoma.

The reported prevalence of TF and TT in Fiji varies significantly between studies. Tests for infection confirm that ocular Ct infections do still occur in Fiji, albeit infrequently and at relatively low load. Our clinical data suggest that trachoma does not meet the WHO definition of a public health problem in this Division of Fiji, but the inconsistency with previous studies warrants further investigation. It is also not clear whether the results from this division will be generalizable to the rest of the country. Recommendations on how best to incorporate this information into trachoma management plans are sparse. Estimating the age-specific prevalence of serological markers for exposure to chlamydial infection [37,38], photographic evidence of phenotype, and exploration of other associations of TF in Fiji could be beneficial in developing those recommendations.

## Supporting Information

S1 Table Indicates the aspects of the study that adhered to the Strengthening the Reporting of Observational Studies in Epidemiology (STROBE) guidelines [39]. (PDF)
