# Supplementary material for: Low Prevalence of Ocular Chlamydia trachomatis Infection and Active Trachoma in the Western Division of Fiji
Source: PLoS Negl Trop Dis. 2016 Jul 12;10(7):e0004798. doi: 10.1371/journal.pntd.0004798 (PMC4942140; doi:10.1371/journal.pntd.0004798)
Supplement: S1 Table — (PDF) [file pntd.0004798.s001.pdf]

Supplementary table 1. STROBE checklist.

	Item No	Recommendation	Completed?	Pre-publication draft page/line
**Title and abstract**	1	(*a*) Indicate the study's design with a commonly used term in the title or the abstract	Yes	Page 2, line 40
		(*b*) Provide in the abstract an informative and balanced summary of what was done and what was found	Yes	Page 2-3
**Introduction**				
Background/rationale	2	Explain the scientific background and rationale for the investigation being reported	Yes	Page 6, line 119-128
Objectives	3	State specific objectives, including any prespecified hypotheses	Yes	Page 6, line 130-133
**Methods**				
Study design	4	Present key elements of study design early in the paper	Yes	Page 6, line 130; Page 8-9, line 156-177
Setting	5	Describe the setting, locations, and relevant dates, including periods of recruitment, exposure, follow-up, and data collection	Yes	Page 7, figure 1; page 8-9, line 156-166
Participants	6	(*a*) Give the eligibility criteria, and the sources and methods of selection of participants	Yes	Page 8-9; line 163-166
Variables	7	Clearly define all outcomes, exposures, predictors, potential confounders, and effect modifiers. Give diagnostic criteria, if applicable	Yes	Page13, line 256-261; page 10, line 191-193; page 11, line 235; page 15, line 300
Data sources/ measurement	8*	For each variable of interest, give sources of data and details of methods of assessment (measurement). Describe comparability of assessment methods if there is more than one group	Yes	Page13, line 256-261; page 10, line 191-193; page 11, line 235; page 15, line 300
Bias	9	Describe any efforts to address potential sources of bias	Yes	Page13, line 256-61
Study size	10	Explain how the study size was arrived at	Yes	Page 9, line 170-177
Quantitative variables	11	Explain how quantitative variables were handled in the analyses. If applicable, describe which groupings were chosen and why		
Statistical methods	12	(*a*) Describe all statistical methods, including those used to control for confounding	Yes	Page13, line 256-61
		(*b*) Describe any methods used to examine subgroups and interactions	Yes	Page13, line 256-61
		(*c*) Explain how missing data were addressed	No	Page 19, line 398-404
		(*d*) If applicable, describe analytical methods taking account of sampling strategy	Yes	Page 9, line 170-177
		(*e*) Describe any sensitivity analyses	Not applicable	-
**Results**				
Participants	13*	(a) Report numbers of individuals at each stage of study—eg numbers potentially eligible, examined for eligibility, confirmed eligible, included in the study, completing follow-up, and analysed	Yes	Page 14, line 268-274
		(b) Give reasons for non-participation at each stage	Not recorded	-
		(c) Consider use of a flow diagram	No	-
Descriptive data	14*	(a) Give characteristics of study participants (eg demographic, clinical, social) and information on exposures and potential confounders	Yes	Page 14, line 268-274
		(b) Indicate number of participants with missing data for each variable of interest	Yes	Page 14, line 268-274; page 14, line 278-279;

				page 15, line 297-299
Outcome data	15*	Report numbers of outcome events or summary measures	Not applicable	
Main results	16	(*a*) Give unadjusted estimates and, if applicable, confounder-adjusted estimates and their precision (eg, 95% confidence interval). Make clear which confounders were adjusted for and why they were included	Yes	Page 14, line 278-288
		(*b*) Report category boundaries when continuous variables were categorized	Not applicable	-
		(*c*) If relevant, consider translating estimates of relative risk into absolute risk for a meaningful time period	Not applicable	-
Other analyses	17	Report other analyses done—eg analyses of subgroups and interactions, and sensitivity analyses	Not applicable	-
**Discussion**				
Key results	18	Summarise key results with reference to study objectives	Yes	Page 17-20
Limitations	19	Discuss limitations of the study, taking into account sources of potential bias or imprecision. Discuss both direction and magnitude of any potential bias	Yes	Page 17-20
Interpretation	20	Give a cautious overall interpretation of results considering objectives, limitations, multiplicity of analyses, results from similar studies, and other relevant evidence	Yes	Page 17-20
Generalisability	21	Discuss the generalisability (external validity) of the study results	Yes	Page 19-20
**Other information**				
Funding	22	Give the source of funding and the role of the funders for the present study and, if applicable, for the original study on which the present article is based	Yes	Journal additional information
